# Sedative and Hypnotic Activities of the Methanolic and Aqueous Extracts of *Lavandula officinalis* from Morocco

**DOI:** 10.1155/2012/270824

**Published:** 2011-11-20

**Authors:** Rachad Alnamer, Katim Alaoui, El Houcine Bouidida, Abdelaziz Benjouad, Yahia Cherrah

**Affiliations:** ^1^Laboratory of Genetic Immunology and Biochemistry, Department of Biology, Faculty of Science, Mohammed V Agdal University, BP 6203, Rabat Instituts, Agdal, Rabat, Morocco; ^2^Laboratory of Pharmacology and Toxicology, Department of Drugs Sciences, Faculty of Medicine and Pharmacy, Mohammed V Souissi University, ERTP, BP 6203, Rabat Instituts, Agdal, Rabat, Morocco; ^3^National Laboratory of Drugs Controlled, BP 6203, Rabat Instituts, Agdal, Rabat, Morocco

## Abstract

We evaluate the sedative and hypnotic activities of the methanolic and aqueous extract of *Lavandula officinalis* L. on central nervous system (CNS). In this study, the effect of the methanolic and aqueous extracts of this plant was investigated in a battery of behavioural models in mice. Stems and flowers of *Lavandula officinalis* L. have several therapeutic applications in folk medicine in curing or managing a wide range of diseases, including insomnia. The methanolic extract produced significant sedative effect at the doses of 200, 400, and 600 mg/kg (by oral route), compared to reference substance diazepam (DZP), and an hypnotic effect at the doses of 800 and 1000 mg/kg while the treatment of mice with the aqueous extract at the doses of 200 and 400 mg/kg via oral pathway significantly reduced in both the reestablishment time and number of head dips during the traction and hole-board tests. In conclusion, these results suggest that the methanolic and aqueous extracts of *Lavandula officinalis* possess potent sedative and hypnotic activities, which supported its therapeutic use for insomnia.

## 1. Introduction

Morocco is fortunate to have such varied climate that almost any medicinal plant can grow. The varied climate and heterogeneous ecologic condition in Morocco have favoured the proliferation of more than 42,000 species of plants, divided into 150 families and 940 genuses [1–3]. Insomnia defined as persistent difficulty in falling or staying a sleep that affects function can induce significant psychological and physical disorder. Sedatives are drugs that decrease activity and have a calming, relaxing effect. At higher doses, sedatives usually cause sleep. Drugs used mainly to cause sleep are called hypnotics. The difference between sedatives and hypnotics, then, is usually the amount of the dose; lower doses have a calming effect and higher doses cause sleep [4]. Recent studies have shown that herbal drugs exert good sedative and hypnotic effect on the central nervous system [4–6]. In recent years, *Lavandula officinalis* flowers exhibit such various biological and pharmacological activities as anti-tumour, anti-inflammatory, antihistaminic, antidiabetic, and antimicrobial activity and modulating the central nervous system [2, 7–11]. The aim of this experiment is to evaluate the sedative and hypnotic activities of *Lavandula officinalis *methanolic and aqueous extract, and to, therefore, determine the scientific basis for its use in traditional medicine in the management of central nervous system disorders.

## 2. Materials and Methods

### 2.1. Plant Material

Stems and flowers of *Lavandula officinalis *L. were collected based on ethnopharmacological information from the villages around the region Rabat-Sale-Zemour-Zaers, with the agreement from the authorities and respecting the United Nations Convention of Biodiversity and with assistance of traditional medical practitioner. The plant was identified with botanist of scientific institute (Pr. M. Ibn Tatou). A voucher specimen (N°256) was deposited in the Herbarium of Botany Department of the Scientific Institute of Rabat.

### 2.2. Preparation of Extract

Stems and flowers of *Lavandula officinalis *were successively extracted with methanol by maceration at room temperature (25°C) over period of 48 hours. 500 g of plant material and one litre of methanol were used in the extraction. Methanol containing the extract was then filtered through Whatman paper and the solvent was vacuum distilled at 65°C in a rotary evaporator. The remaining extract was finally dried in the oven at 30°C for two hours to ensure the removal of any residual solvent (lyophilisation). Final extract was a dark green powder in percentage dry weight 21.8%. For the aqueous extract, 500 g of plant material was extracted by infusion boiled water (500 mL) for three days. The respective aqueous extracts were separated from its residues by gravity filtration. The final crude extract was obtained as yellow greasy powder in percentage from dry weight (15.7% d.w). These extracts were kept in deep freeze at −20°C until use.

### 2.3. Animals

Male Swiss mice (20–25 g) (Iffa-credo, France) were used in pharmacological tests and females of the same strain in the LD_50_ calculation. The animals were fed *ad libitum *with standard food and water except when fasting was required in the course of the study. The animals were acquired from the animal experimental centre of Mohammed V Souissi University, Medicine and Pharmacy Faculty, Rabat.

### 2.4. Acute Toxicity

Median lethal dose (LD_50_) values were determined as described by Litchfield and Wilcoxon [12]. Seven groups of mice of both sexes (*n* = 10, 5 males and 5 females) received or not single oral doses at different concentrations (500, 1000, 1500, 2000, 3000, and 5000 mg/kg, p.o.). The control group received only the water or saline solution. After a single dose administration, mice were placed in individual clear plastic boxes and continuously observed for 6 h and at 24 h time interval to detect any eventual side effects. The number of animals, which died during this period, was expressed as percentile. The LD_50_ of the extract were estimated by the p.o. route using the procedure reported by Litchfield and Wilcoxon; the method estimated the dose of the extract that would kill 50% of a reduced sample of animals by a given route. In a first phase, the extract was given to ten mice per group at doses of 500 and 1000 mg/kg; when no mortality was observed, the doses were increased to 1500, 2000, 3000, and 5000 mg/kg. Mice were kept under observation for 14 days to register possible mortality, their weights were registered, and at the end of the study they were sacrificed for macroscopic tissue examination [13], and the LD50 was determined by probit test using death percent versus doses log [14–16]. Of note, drugs used as control were given to mice in similar conditions.

### 2.5. Drugs

All drugs and extracts were freshly prepared on the day of the experiments. A control group received distilled water (10 mL/kg, p.o.) as vehicle. Diazepam (3 mg/kg, i.p., a conventional sedative) and thiopental (60 mg/kg, i.p., a conventional hypnosis) were used as positive control.

### 2.6. Pharmacological Evaluations

The activity of methanolic and aqueous extract from *Lavandula officinalis* on the central nervous system was then studied, using a battery of behavioral tests used in psychopharmacology. We analyzed the effect of different doses of the methanolic extracts (100, 200, 400, and 600 mg/kg, p.o.) and aqueous extracts (100, 200, and 400 mg/kg, p.o.) from Lavandula officinalis for their sedative and hypnotic activities. For testing sedative effect, the effect of extract on mice was qualified in one of the following tests. 

### 2.7. Traction Test

Mice were individually suspended by anterior limbs to a wire stretched horizontally. Abnormal mice that fail to make a reestablishment at least one of its posterior limbs to reach the wire are considered as subject under a sedative action. When the animals perform normal reestablishment immediately, the reaction is known as positive; other wise, the reaction is called negative; also, the behaviours of animals were recorded during the period of the experiment [17, 18].

### 2.8. Fireplace Test

The apparatus used for this test consist of a vertical glass tube 30 cm in length. Mice were individually placed vertically in the glass test tube, a normal mouse typically attempts to escape in thirty seconds, and the mice considered as subject to the sedative effect when performing the rise of cylinder greater than 30 sec [19].

### 2.9. Hole-Board Test

Mice were individually placed in the centre of a perforated board, and the number of head dips was registered during a 5 min. The perforated board test was made by using a wood floor board, 40 cm × 40 cm × 25 cm, in which evenly spaced holes were made. The number of explored holes provide a measure of the number of head dips [20, 21].

### 2.10. Thiopental-Induced Sleep in Mice

Thiopental (a sub-hypnotic dose) 60 mg/kg was injected i.p. 30 min after administration of methanolic and aqueous extracts. The mice were treated with different doses of methanolic and aqueous extracts (800, 1000 mg/kg, p.o., *n* = 5), the control group (*n* = 5) was treated with distilled water (10 mL/kg, p.o.), and positive control group (*n* = 5) was administrated with diazepam (3 mg/kg, i.p.), respectively. The effect was recorded for disappearance (latency) and reappearance (duration) of the righting reflex. Hypnotic sleeping time was considered to be the time interval between disappearance and reappearance of the righting reflex [5, 22].

### 2.11. Statistical Analysis

The statistical analysis was done using ANOVA. The results with *P* < 0.05 were considered significant. The data are expressed as mean ± SD.

## 3. Results

### 3.1. Acute Toxicity of *Lavandula officinalis* Methanolic and Aqueous Extracts

Following oral administration of *Lavandula officinalis *extract at the doses of 500, 1000, 1500, 2000, 3000, and 5000 mg/kg, p.o., no toxicity and no significant changes in the body weight between the control and treated group were demonstrated at these doses. This result indicates that, the LD_50_ was higher than 5000 mg/kg.

### 3.2. Sedative Activity of the Methanolic and Aqueous Extract on the Central Nervous System (CNS)

The results of psychotropic effects of methanolic and aqueous extracts were expressed by comparison with control groups. Pharmacological tests were then performed at nontoxic doses (i.e., 100, 200, 400, and 600 mg/kg, p.o.), for the methanolic extract and (100, 200, and 400 mg/kg, p.o.), for the aqueous extract.

### 3.3. Traction Test

The methanolic extract of *Lavandula officinalis *given by oral route at 100 mg/kg did not significantly alter the reestablishment time; all animals performed normal reestablishment immediately (*P* > 0.05). However, the extract at the dose of 200 mg/kg produced significant sedative effect on the central nervous system (CNS) as indicated by the relatively high time for the reestablishment of the mice (Table 1). By increasing the doses to 400 and 600 mg/kg, the average reestablishment time was increased. The reestablishment time was notably higher than control group (*P* < 0.001) (Table 1). For the aqueous extract, after oral administration at the dose of 100 mg/kg, all animals performed normal reestablishment time (notably decreased the reestablishment time). This indicates that this extract produced no significant sedative effect on mice behavior at this dose (*P* > 0.05) (Table 2). By increasing the doses to 200 and 400 mg/kg, the reestablishment time was increased; the mice fail to make a reestablishment immediately (*P* < 0.001). So, the aqueous extract of *Lavandula officinalis *produced significant sedative effect at the doses of 200 and 400 mg/kg p.o. (Table 2). In addition, the dose of 100 mg/kg of both extracts did not decreased the reestablishment time.

### 3.4. Fireplace Test

Animal treated with the methanolic extract of *Lavandula officinalis *at the dose of 100 mg/kg via oral route do not show loss of initiative and curiosity. By increasing the doses to 200, 400, and 600 mg/kg, all mice lose initiative and curiosity; that is, animal did not attempt to mount the tube for escape (*P* < 0.01) (Table 1). While, the aqueous extract, at the doses of 100 mg/kg, produced no sedative effect, at the doses of 200 and 400 mg/kg p.o., all animals treated showed loss of initiative and curiosity (*P* < 0.001) (Table 2).

### 3.5. Hole-Board Test

In the hole-board test, a significant reduction in the number of head dips at the doses of 200, 400, and 600 mg/kg by oral route administration; with the exception at the dose of 100 mg/kg, the methanolic extract did not reduce the number of head dips. However, animal treated with the aqueous extract at the doses of 100 mg/kg did not reduce the number of head dips (*P* > 0.05). By increasing the doses to 200 and 400 mg/kg, this extract reduced the cumulative number of holes explored and the number of spaces between two holes explored (en relation with motor activity) (*P* < 0.001) (Table 1). The data lead to conclude that the methanolic and aqueous extract of *Lavandula officinalis* possess potential sedative effects on the central nervous system at the doses of 200, 400, and 600 mg/kg via oral route administration (Tables 1 and 2).

### 3.6. Thiopental-Induced Sleep in Mice

We observed that the lavender methanolic extract (ME) had induced hypnotic effect significant (*P* < 0.001). Hypnosis induced by methanolic extract (800 and 1000 mg/kg, p.o.) was evaluated by observation of the duration of thiopental-induced sleeping time. The extract showed a reduction in the time of onset of sleep induced by thiopental. The effects of the extract on onset of sleep at 800 and 1000 mg/kg were comparable to that of diazepam at 3 mg/kg. The highest prolongation of sleep produced by the methanolic extract was comparable to that of diazepam (3 mg/kg). Only the highest dose tested for ME significantly increased from 45 ± 2  to 112 ± 3 min; the duration of hypnos is induced by thiopental (Table 3). However, the aqueous extract of *Lavandula officinalis *at the doses of 800 and 1000 mg/kg, p.o., produced no hypnotic activity significant on the central nervous system confirming, thus, the hypnotic action of lavender methanolic extract at high doses by oral pathway (Table 3).

## 4. Discussion

In aromatherapy, the methanolic and aqueous extracts of *Lavandula officinalis *are believed to possess anticonvulsive, sedative, hypnosis, and antidepressive effects and to be useful for treating nervous breakdown, nervous tension, depression, and insomnia [23–25]. In this paper, we observed the sedative and hypnotic properties of methanolic and aqueous extract from *Lavandula officinalis *L. in mice. Therefore, in order to study the comprehensive effect of our drugs, the following targets were observed: reestablishment time, number of head dips, and loss of initiative and curiosity in mice.

Diazepam is central nervous system depressant used in the management of sleep disorders such as insomnia; these compounds have a binding site on GABA receptor type-A ionophore complex (GABA_A_) [4, 5]. It decreases activity, moderates excitement, and calms the recipient. Substances like diazepam (which has been chosen as the standard reference drug in this study) reduce onset of and increase duration of barbiturate-induced sleep and reduce exploratory activity possessing potentials as sedative [21, 26]. Lavender extract increased the time of reestablishment by mice in the traction test (Table 1), after oral administration of 200, 400, and 600 mg/kg dosages, producing sedative effect similar to that observed with 3 mg/kg diazepam. Diazepam is a very well-known anxiolytic benzodiazepine (BDS) which produces not only anxiolytic-like effect but also important sedative action. In this respect, lavender extract produced a dose-dependent reduction in the number of head dips in the hole-board test similar and/or greater than diazepam (Tables 1 and 2). It is generally believed that locomotor activity results from brain activation, which is manifested as an excitation of central neurons involving different neurochemical mechanism and an increase in cerebral metabolism. It is possible that the sedative activity of methanolic and aqueous extract of Lavandula officinalis is mediated by GABAergic pathway, since GABAergic transmission can produce profound sedation in mice [27]. The inhibitory action of GABA consists in the opening of chloride channels to allow hyperpolarizing the membrane, leading to CNS depression and resulting in sedative and hypnosis activity. Glutamate and GABA are quantitatively the most important excitatory and inhibitory neurotransmitters, respectively, in the mammalian brain [28]. Thus, receptors for these two neurotransmitters are regarded as important targets for psychotropic drugs. In the test of thiopental-induced sleep in mice, the potentiated effect of lavender extract in mice was represented. It not only prolonged the sleeping time but also decreased the latency of falling asleep and increases the rat of sleep onset. The lavender extract has produced hypnosis at high doses' that is, 800 and 1000 mg/kg. Since the effect of thiopental on the CNS involves the activation of the inhibition GABAergic system [29, 30], this finding suggests that some constituents in lavender extract produce facilitation of this inhibitory system (Table 3). Phytochemical studies have identified active components in this plant such as coumarin, chalcones, flavanones, flavones, flavonols, quercetin, and kaempferol derivatives, suggesting that they are the main responsible for sedative and hypnotic activities [4, 6, 31]. Further chemical and pharmacological analysis of the extract will be conducted to isolate and characterize the active principles responsible for the sedative and hypnotic effect. In conclusion, p.o. administration of methanolic and aqueous extract of Lavandula officinalis induces similar sedative effects, supporting its use in folk medicine. Given that the LD_50_ value for these extracts was beyond 5000 mg/kg for oral administration, as determined by Litchfield and Wilcoxon [12]; our results suggest a remote risk of acute toxicity and good tolerance of these extracts in traditional medicine. To sum up, this work represents that the methanolic and aqueous extracts of *Lavandula officinalis* have obvious sedative and hypnotic activity; these data provide pharmacological basis for its therapeutic efficacy on insomnia.

## Figures and Tables

**Table 1:** Sedative action of *Lavandula officinalis *methanolic extract. p.o. means oral route; i.p. means intraperitoneal route; *n* means number of mice per group; sec means seconds; ME: mean methanolic extract; DZP means diazepam. Data are expressed as mean ± SD; *P* < 0.001 versus the control group.

Test		Control	Diazepam i.p.	Methanolic extract of *Lavandula officinalis *in mg/kg p.o.			
			DZP (3 mg/kg)	ME (100 mg/kg)	ME (200 mg/kg)	ME (400 mg/kg)	ME (600 mg/kg)
Traction test	Re-establishment time	0.09 sec ± 0.0	10 sec ± 0.3	0.08 sec ± 0.5	5 sec ± 0.5*	18 sec ± 0.5*
		*n* = 5	*n* = 5	*n* = 5	*n* = 5	*n* = 5
Fireplace test	Time to go back the tube in seconds	7 sec ± 0.5	>2 min	sec ± 0.5	30 sec ± 1*	0.58 sec ± 1*
		*n* = 5	*n* = 5	*n* = 5	*n* = 5	*n* = 5
Hole-board test	Explored holes during 5 minutes	10 ± 1	0.0 ± 0.0	8 ± 0.1	2 ± 0.0*	1 ± 0.0*
		*n* = 5	*n* = 5	*n* = 5	*n* = 5	*n* = 5

**Table 2:** Sedative effect of aqueous extract of *Lavandula officinalis. *p.o. means oral route; i.p. means intraperitoneal route; *n* means number of mice per group; sec means seconds; AE means aqueous extract; DZP means diazepam. Data are expressed as mean ± SD; *P* < 0.001 versus the control group.

Test		Control	Diazepam i.p.	Aqueous extract of *Lavandula officinalis *p.o.		
			DZP (3 mg/kg)	AE (100 mg/kg)	AE (200 mg/kg)	AE (400 mg/kg)
Traction test	Re-establishment time	0.09 sec ± 0.0	10 sec ± 0.3	2 sec ± 0.3	12 sec ± 0.5*
		*n* = 5	*n* = 5	*n* = 5	*n* = 4
Fireplace test	Time to go back the tube in seconds	7 sec ± 0.5	>2 min	12 sec ± 0.1	45 sec ± 1*
		*n* = 5	*n* = 5	*n* = 5	*n* = 5
Hole-board test	Explored holes during 5 minutes	10 ± 1	0.0 ± 0.0	6 ± 0.2	1 ± 0.0*
		*n* = 5	*n* = 5	*n* = 5	*n* = 4

**Table 3:** Effect of the methanolic extract of *Lavandula officinalis *on the onset and duration of sleep in thiopental-treated mice. Mice received thiopental (60 mg/kg, i.p.) 30 min after the pretreatment of methanolic extract (800 and 1000 mg/kg, p.o.) and diazepam (3 mg/kg, i.p.). i.p. means intraperitoneal route; p.o. means oral route; (*n* = 5) means number of mice per group; ME means methanolic extract; D ZP means diazepam. Data are expressed as mean ± SD; *P* < 0.001 versus the control group.

Group	Dose (mg/kg)	Sleep latency (min)	Sleeping time (min)
Normal	60	8 ± 1	36 ± 3
DZP	3	6 ± 1*	75 ± 3*
ME	800	12 ± 0.5*	45 ± 2*
ME	1000	6 ± 1*	112 ± 3*
