# Corrigendum to “Sedative and Hypnotic Activities of the Methanolic and Aqueous Extracts of *Lavandula officinalis* from Morocco”

**DOI:** 10.1155/2020/5695093

**Published:** 2020-11-12

**Authors:** Rachad Alnamer, Katim Alaoui, El Houcine Bouidida, Abdelaziz Benjouad, Yahia Cherrah

**Affiliations:** ^1^Laboratory of Genetic Immunology and Biochemistry, Department of Biology, Faculty of Science, Mohammed V Agdal University, BP 6203, Rabat Instituts, Agdal, Rabat, Morocco; ^2^Laboratory of Pharmacology and Toxicology, Department of Drugs Sciences, Faculty of Medicine and Pharmacy, Mohammed V Souissi University, ERTP, BP 6203, Rabat Instituts, Agdal, Rabat, Morocco; ^3^National Laboratory of Drugs Controlled, BP 6203, Rabat Instituts, Agdal, Rabat, Morocco

In the article titled “Sedative and Hypnotic Activities of the Methanolic and Aqueous Extracts of *Lavandula officinalis* from Morocco” [1], there was a substantial amount of material from previously published articles, including the following sources cited as [4, 6, 31]:

- F. Huang, Y. Xiong, L. Xu, S. Ma, and C. Dou, “Sedative and hypnotic activities of the ethanol fraction from Fructus Schisandrae in mice and rats,” Journal of Ethnopharmacology, vol. 110, no. 3, pp. 471–475, 2007. [2].
- G. Pérez-Ortega, P. Guevara-Fefer, M. Chávez et al., “Sedative and anxiolytic efficacy of *Tilia americana* var. *mexicana* inflorescences used traditionally by communities of State of Michoacan, Mexico,” Journal of Ethnopharmacology, vol. 116, no. 3, pp. 461–468, 2008. [3].
- G. Zapata-Sudo, T. C. F. Mendes, M. A. Kartnaller et al., “Sedative and anticonvulsant activities of methanol extract of *Dorstenia arifolia* in mice,” Journal of Ethnopharmacology, vol. 130, no. 1, pp. 9–12, 2010. [4].
- Additionally, the article was also found to contain a substantial amount of material, without citation, from previously published articles, including the following sources:
- E. Aguirre-Hernández, A. L. Martínez, M. E. González-Trujano, J. Moreno, H. Vibrans, M. Soto-Hernández, “Pharmacological evaluation of the anxiolytic and sedative effects of *Tilia americana* L. var. *mexicana* in mice,” Journal of Ethnopharmacology, vol. 109, no. 1, 2007. [5].
- O. O. Adeyemi, A. J. Akindele, O. K. Yemitan, F. R. Aigbe, F. I. Fagbo, “Anticonvulsant, anxiolytic and sedative activities of the aqueous root extract of *Securidaca longepedunculata* Fresen.,” Journal of Ethnopharmacology, vol. 130, no. 2, 2010. [6].

## References

[1] Alnamer R., Alaoui K., Bouidida E. H., Benjouad A., Cherrah Y. (2012). Sedative and hypnotic activities of the methanolic and aqueous extracts of *Lavandula officinalis* from Morocco. *Advances in Pharmacological Sciences*.

[2] Huang F., Xiong Y., Xu L., Ma S., Dou C. (2007). Sedative and hypnotic activities of the ethanol fraction from Fructus Schisandrae in mice and rats. *Journal of Ethnopharmacology*.

[3] Pérez-Ortega G., Guevara-Fefer P., Chávez M. (2008). Sedative and anxiolytic efficacy of *Tilia americana* var. *mexicana* inflorescences used traditionally by communities of State of Michoacan, Mexico. *Journal of Ethnopharmacology*.

[4] Zapata-Sudo G., Mendes T. C. F., Kartnaller M. A. (2010). Sedative and anticonvulsant activities of methanol extract of *Dorstenia arifolia* in mice. *Journal of Ethnopharmacology*.

[5] Aguirre-Hernández E., Martínez A. L., González-Trujano M. E., Moreno J., Vibrans H., Soto-Hernández M. (2007). Pharmacological evaluation of the anxiolytic and sedative effects of *Tilia americana* L. var. *mexicana* in mice. *Journal of Ethnopharmacology*.

[6] Adeyemi O. O., Akindele A. J., Yemitan O. K., Aigbe F. R., Fagbo F. I. (2010). Anticonvulsant, anxiolytic and sedative activities of the aqueous root extract of *Securidaca longepedunculata* Fresen. *Journal of Ethnopharmacology*.

